# Rationale: Photosynthesis of Vascular Plants in Dim Light

**DOI:** 10.3389/fpls.2020.573881

**Published:** 2020-11-23

**Authors:** Xiaolin Wang, Yong Wang, Aifeng Ling, Zhen Guo, Muhammad Asim, Fupeng Song, Qing Wang, Yanguo Sun, Rayyan Khan, Huifeng Yan, Yi Shi

**Affiliations:** ^1^Tobacco Research Institute, of Chinese Academy of Agricultural Sciences, Qingdao, China; ^2^Liangshan Branch of Sichuan Tobacco Company, Xichang, Qingdao, China; ^3^First Institute of Oceanography, Ministry of Natural Resources, Qingdao, China; ^4^College of Resources and Environment, Shandong Agricultural University, Tai’an, China; ^5^College of Tropical Crop, Hainan University, Haikou, China

**Keywords:** dim light, light harvest, stomatal behavior, light induction, carbon consequence, photosynthesis

## Abstract

Light dominates the earth’s climate and ecosystems via photosynthesis, and fine changes of that might cause extensive material and energy alternation. Dim light (typically less than 5 μmol photons m^–2^ s^–1^) occurs widely in terrestrial ecosystems, while the frequency, duration, and extent of that are increasing because of climate change and urbanization. Dim light is important for the microorganism in the photosynthetic process, but omitted or unconsidered in the vascular plant, because the photosynthesis in the high-light adapted vascular leaves was almost impossible. In this review, we propose limitations of photosynthesis in vascular plant leaves, then elucidate the possibility and evidence of photosynthesis in terms of energy demand, stomatal opening, photosynthetic induction, and photosynthesis-related physiological processes in dim light. This article highlights the potential and noteworthy influence of dim light on photosynthesis in vascular plant leaves, and the research gap of dim light in model application and carbon accounting.

## Introduction

Plants use light both as a source of energy via photosynthesis and as a source of information (Gaston et al., 2013; Bennie et al., 2016). Leaves are always exposed to the environment with fluctuating light, which rapidly shift from being limiting for photosynthesis to high levels (Retkute et al., 2015). Dim light (typically less than 5 μmol photons m^–2^ s^–1^) is universal in natural and artificial ecosystems, such as twilight, dawn, and moonlight (Salisbury, 1981; Raven and Cockell, 2006; Bennie et al., 2016), deep ocean (Dubinsky and Schofield, 2010; Ezequiel et al., 2015), understory (Pearcy, 1983; Valladares et al., 2011), and artificial night illumination (Gaston et al., 2013; Bennie et al., 2016; Davies and Smyth, 2018; Table 1). Some plants switch light conditions among different intensities. In most cases, dim light is considered as useless light for net carbon fixation, because the levels of photosynthetically active radiation (PAR) are far below the sunlit conditions (between 100 and 2000 μmol photons m^–2^ s^–1^).

**TABLE 1:** Light intensity of some types of dim light environment.

Light conditions	PPFD (μ mol photons m^–2^ s^–1^)	Data sources
Earth surface at the full moon	0.004	Breitler et al., 2020
300 m below the sea surface	0.02	Raven et al., 2000
Understory of rainforest	0.1	Pearcy et al., 1985
The average intensity of urban light pollution	0.5–1	Gaston et al., 2013
100 W-incandescent lamp	3	Measured in 5-m distance
150 W-fluorescent lamp	5	Measured in 5-m distance

*The average light intensity of the incandescent lamp and the fluorescent lamp was measured with an optic spectrometer (AvaSpec-ULS2048XL, Avantes, Netherlands) in Beijing (39°28′N- 41°05′N–115°25′E-117°35′E), China, *N* = 100.*

However, dim light is an exclusive energy source for photosynthesis in some species associated with dim light over a long period of time, for instance, algae and photolithotrophs in the oceans must harvest the very low light to drive photosynthesis because the PAR below the sea surface is greatly decreased, especially in the deep ocean (Dubinsky and Schofield, 2010; Ezequiel et al., 2015). Dim light possibly fulfills the energy demand for the metabolism of a unicellular organism, hence, playing important roles in marine life and marine carbon sequestration. For the multicellular green plants, the use of dim light is also crucial when they are exposed in a dim environment. For example, understory plants have to acclimate as low as 0.1 μmol photons m^–2^ s^–1^ PPFD and complete their lifecycles (Salisbury, 1981; Pearcy, 1983; Valladares et al., 2011; Ezequiel et al., 2015). In actuality, dim light has been more common in recent decades due to the decrease in radiation reaching the earth surface with rising atmospheric aerosol, caused by anthropogenic emissions (Mercado et al., 2009), thus the areas of low light expanded. For another case, increasing urbanization has changed a large area of natural lands to urban lands, which would suffer great shade by urban structures in the daytime and multitudinous light pollution in nighttime (Gerrish et al., 2009; Gaston et al., 2013). To the best of our knowledge, the estimation of carbon sequestration in terrestrial ecosystems failed to take CO_2_ assimilation of green plants in dim light into consideration, particularly in urban areas. This might be caused by inconclusive effects of dim light on the CO_2_ assimilation sequestration, and the global carbon sequestration needs to be given wide attention.

The photosynthesis in vascular plant leaves is determined not only by energy demand but also stomatal opening and activity of a biochemical enzyme (Rubisco), which is greatly affected by PPFD. The energetic demands for photosynthesis in the vascular green plant were quite different from unicellular organisms. The photosynthesis in dim light in the unicellular organisms was widely investigated, but there were no findings about the response of photosynthetic light reaction and dark reaction to dim light in higher green plants. One of the noticeable problems is whether the high-light adapted vascular plant leaves could take full advantage of dim light for photosynthesis, because the vascular plant leaves need to capture the light and CO_2_ passing through the epidermis, cytoderm, cytomembrane, and activate the necessary light-dependent photosynthetic enzymes.

In this review, we presented the limitation of photosynthesis in leaves of the vascular plants and explained the possibility of photosynthesis in terms of the driving force of reaction, stomatal opening, and activation of biochemical reaction in dim light. We critically appraised the evidence of great importance of the dim light in photosynthesis in a vascular plant and emphasized the importance of comprehensive re-consideration to those processes in photosynthetic ecophysiology and carbon sequestration of terrestrial ecosystems.

## Photosynthesis in Dim Light

### Limitation of Photosynthesis of Vascular Plants in Dim Light

The lowest photon flux density of PAR at which O_2_-evolving photolithotrophs on earth appears to be able to generate photosynthesis is 10 nmol photons m^–2^ s^–1^ (Raven et al., 2000). In addition, Quigg et al. (2003) had also proved that the protein turnover, charge recombination in PSII, and proton leakage and slippage of *Dunaliella tertiolecta* and *Phaeodactylum tricornutum* could generate in dim light, respectively in the value of 30 and 3 μmol photons m^–2^ s^–1^ (Quigg et al., 2003). Photosynthesis in plant cells occurs in the chlorophyll-containing chloroplast and assimilates CO_2_ in photosynthetic apparatus (Singhal et al., 2019), whether the initiation of the photosynthesis will mainly depend on the driving force of photoreaction, the capacity of CO_2_ supply, and the activity of photosynthetic apparatus. Thus, energy demand, stomatal behavior, and induction of photosynthetic apparatus are the key limitations of photosynthesis under dim light conditions.

### The High Energy Transfer Efficiency in Photochemical Systems of Plant Leaves

Plants have a large variety of light-harvesting strategies to adapt nearly everywhere sunlight can penetrate. The interaction of two photosynthetic pigments was synergistic on light harvesting and the absorbed light energy from plenty of antenna pigments focuses on one reaction center (RC) pigment. One typical RC and the surrounded about 250–300 pigment molecules comprise a functional photosynthetic unit (PSU) (Croce and Van Amerongen, 2014). The number of chloroplasts is kept in steady state among most of the plant species (Kura-Hotta et al., 1990; Ono et al., 1995), ranging from tens to hundreds, and thus the total surface areas of chloroplasts are greatly higher than a leaf area. The chloroplast is a typical spheroid with 5–10 μm long axis containing 10^9^ chlorophyll molecules per chloroplast (Melis et al., 1998). Therefore, an enormous amount of pigment molecules produces effective energy conversion and transformation in plant leaves, resulting an efficient light harvesting.

**FIGURE 1:**
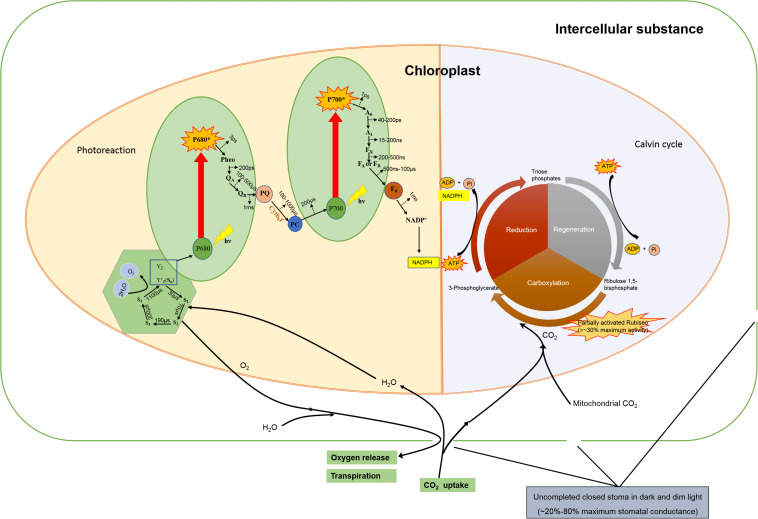
A diagram for linear electron transfer pathway, Rubisco activities and stomatal behavior of a vascular plant leaf in the dim light. The references were showed in the “Supplementary Material”. P680: reaction center pigment molecules of PSII, P680^+^: oxidation state of P680, P680^∗^: excited state of P680, pheo: pheophytin, Q_*A*_ and Q_*B*_: plastoquinone, Cytb_6_f: cytocrom b6f complex, PQ: quinine sink, PC: plastocyanin, P700: reaction center pigment molecules of PSI, P700^+^: oxidation state of P700, P700^∗^: excited state of P700, A_0_: the primary electron acceptor of PSI, A_1_: the secondary electron acceptor of PSI, F_x_(FA,FB): iron-sulfur cluster, F_d_: ferredoxin, Y_z_: tyrosine residues of D_1_ protein, S_0_(S_1_, S_2_, S_3_, S_4_): redox state.

The RC would be inactive without an antenna, the capacity of light-harvesting is crucial, especially in light-limited conditions (Croce and Van Amerongen, 2014). Thus in such conditions the antenna pigments transfer their excitation energy typically within 1 ps to Chl a, and the excitation energy transfer proceeds via Chl a. The arrival of excitation in the RC, typically within 10^–10^ s after initial photon capture by the antenna, leads to efficient electron transfer from a primary donor (P680 of PSII) to the primary acceptor. Upon excitation by light P680 in PSII causes charge separation and releases an electron, which initiates the linear electron transfer pathway, and P680 turns to an excited state (P680^∗^). The electron eventually leads to the reduction of the primary donor P700 of PSI, which is oxidized after it has donated an electron to Fd after light excitation through pheophytin (pheo, 3 ps), plastoquinone (Q_*A*_ and Q_*B*_, 200 ps), cytochrome b_6_f complex (Cytb_6_f), quinine sink (PQ), and plastocyanin (PC, 100 to 1000 μs). The period of electron transfer from P680 to P700 takes less than 2 ms calculated from the most time-consuming process (Q_*A*_ to PC). The electron in P700^∗^ transfers via two electron acceptors (A_0_ and A_1_) and iron-sulfur cluster (F_*x*_, F_*A*_, and F_*B*_) to ferredoxin (F_*d*_), and finally delivers to oxidized nicotinamide adenine dinucleotide phosphate (NADP^+^). This process approximately takes 100 μs. P680^+^ is one of the products of charge separation, which can be reduced by a tyrosine residue (Y_*z*_) in 20–260 ns, and finally be reduced by manganese cluster in 30–1000 μs via state S_1_ to state S_4_ (Kok et al., 1970; Dismukes and Siderer, 1981). Haumann and Junge (1994) reported that the water oxidation was a millisecond reaction step on transition S_4_ to S_0_, which finally liberated dioxygen (Haumann and Junge, 1994). The half-rise times of four flashes induced the fast release proton were less than 100 μs at pH 7.4 and 6.3 (Haumann and Junge, 1994). The water oxidation takes less than 2 ms to evolve O_2_ on the thylakoid membranes (Mcevoy and Brudvig, 2006). Figure 1 showed that the whole process of light reaction would take 2–3 ms via the two reaction centers (Haumann and Junge, 1994; Mcevoy and Brudvig, 2006).

There is also a risk for efficient light harvesting in restricted time. The pigments cannot remain excited for a long period, and consequently the energy will be lost as heat, radiation, or in other ways. A delay time recently reported for the PSII antenna in plants is 2 ns (Belgio et al., 2012). Thus, to guarantee a fast enough rate and a high quantum yield, the PSII in plants is mainly organized in a supercomplex (Van Bezouwen et al., 2017; Kouřil et al., 2018). The quantum efficiency of the supercomplex is near 100%, and the delay time is around 0.15 ns (Caffarri et al., 2011). Fast and effective electron transfer prevents quenching and returning of the electron. It has been accepted that the Z scheme for photosynthesis proposed by Hill and Bendall (1960) revealed two photoreaction centers, and each required 4 photons to evolve one molecule of O_2_, and require 8 photons assuming the same energy distribution of two photosystems (Putnam-Evans and Barry, 2007). The energy of a single excited chlorophyll molecule cannot exceed 180 kJ mol^–1^, but the reduction of NADP^+^ (electron transfer from water to NADP^+^) needs the energy of 230 kJ⋅mol^–1^. Thus, there should be plenty of excited chlorophyll molecules to accomplish a certain “climbing step.” The energy needed in reducing CO_2_ into carbohydrate is 470 kJ mol^–1^ (Belgio et al., 2012), approximately equivalent to the energy of 8 photons assuming that the efficiency of multiphoton processes is up to 33%.

Experiments of isolated chloroplasts flashing by Joliot et al. (1969) showed that the dark-adapted chloroplasts fail to evolve O_2_ after two-millisecond flashings, but the most O_2_ could be detected in the third flashing and fourth flashing followed, and after that there was an O_2_-evoluting peak every four flashings (Joliot et al., 1969; Joliot, 2003). The O_2_-evoluting model presented by Kok reveals that the oxygen-evolving complex (OEC) can store one charge after flashing, and four stored charges can be used for water-splitting (Kok et al., 1970). Therefore, the electrons from charge separation can be stored, rather than quenching or shifting immediately.

Thus, a consequence of the dim light leads to the effective charge separation and recombination in plant leaves. This fast and effective electron transfer prevents quenching and returning of the electron, and the electrons for water splitting can be stored temporarily in the manganese clusters. These features provide feasible ATP and NADPH for photosynthesis. And the huge number of RC and antenna increases the probability of the above processes. Theoretically, the photosynthetic photoreaction in plant leaves can be driven by the energy of dim light.

### Stomatal Behavior in the Dark or in Dim Light

Plants require sufficient CO_2_ to enter the leaf for photosynthesis. The stomata are formed from two specialized cells (guard cells) in the epidermis, which are morphologically distinct from general epidermal cells and are responsible for regulating stomatal aperture and gas exchange between plants and atmosphere (Blatt, 2000; Julian et al., 2001). Responses of stomata to light are one of the key factors influencing photosynthesis. Stomatal closure at a low light intensity or in darkness results in reduced water loss when the potential photosynthetic rate is low. The stomata of CAM (Crassulacean acid metabolism) species, such as *Ananas comosus, Agave americana L, Opuntia Tourn. ex Mill, Cymbidium* are closed in daytime but open in the nighttime to adapt to an arid environment (Lee, 2010), and in some C_3_ and C_4_ species, the night-open of stomata were also observed in dark or dim light (Meidner and Mansfield, 1965; Kaufmann, 1976; Grulke et al., 2004; Ogle et al., 2012). The length of the preceding dark period might be more important than light intensity in determining the stomatal opening, which is mainly a behavior of circadian rhythms (Meidner and Mansfield, 1965).

The change in the turgor pressure of the cell causes movement in guard cells, which has been regarded as the major mechanism of blue-light mediated response, whereas the change of the intercellular CO_2_ mediated movements of guard and mesophyll cells has been regarded as a major mechanism for regulation of stomatal aperture by photosynthesis (Kaufmann, 1976; Shimazaki et al., 2007; Wang et al., 2008; Lawson, 2009). It has been reported that the dim intensities of white light, down to 10 lux (the equivalent of about 0.81 μmol photons m^–2^ s^–1^), were found sufficient to induce the response of stomatal nighttime opening (Meidner and Mansfield, 1965; Yamori et al., 2020). The blue light mediated reaction to the stomatal opening can be driven by bioenergy transferred from as low as 3 μmol photons m^–2^ s^–1^ light intensity (Meidner and Mansfield, 1965; Shimazaki et al., 2007). A powerful proof by contradiction is if stomata close in the nighttime, how do they provide oxygen for mitochondrial respiration. Photosynthesis in dim light might be very low, and partial stomatal opening could make standard the CO_2_ demand for photosynthesis. The nocturnal stomatal conductance in C_3_ and C_4_ plants was reported in recent years, which contributes to water loss at night (Hoshika et al., 2018; Resco De Dios et al., 2019). The benefit of the stomatal opening thus remains a confusion for botanists. But from those reports it might suggest that the stoma remain open in dark, let alone generate photosynthesis in dim light (such as moonlight) (Mayoral et al., 2020). The simplest explanation is that plants lack stomatal control during the night, and the stomata remain leaky overnight (Resco De Dios et al., 2019). Thus, the stomata behavior in the dim light fails to present a significant obstacle to carbon assimilation.

### Photosynthetic Induction in the Dark or in Dim Light

The photosynthetic apparatus require an induction after a long period in darkness, ranging from minutes to several hours (Osterhout and Haas, 1918). The induction involves the buildup of ribulose-1,5-bisphosphate (RuBP) in the Calvin cycle, the activation of Rubisco (Pearcy et al., 1985; Pearcy, 1988), and stomatal opening (Han et al., 1999; Schulte et al., 2003). Loss of quantum yield in the dark and dim light is one of the important reasons for induction. The period of low light (includes darkness) and intensity of actinic light has great effects on the period of photosynthetic induction (Kirschbaum et al., 2004). The activation level of Rubisco is determined by pH, intercellular CO_2_ concentration, and Mg^2+^ concentration, but the mechanisms of the activating reaction of Rubisco have not yet been completely understood (Carmo-Silva and Salvucci, 2013).

Despite this, there is still evidence that the Rubisco in leaves is still activated after a long period of darkness. A significant difference in photosynthetic efficiency was observed in street light pollution with the PAR less than 0.5 μmol photons m^–2^ s^–1^ (Meravi and Kumar Prajapati, 2020). The activity of *in vitro* Rubisco in *Raphanus sativus L* leaves in the dark was 30% before light induction (Caemmerer and Edmondson, 1986; Von Caemmerer, 2000). Salvucci et al. (1986) showed that Rubisco in *Arabidopsis thaliana L. Heynh* could remain active after a 60-min darkness, and the activity of Rubisco could quickly rise when exposed to low light (Salvucci et al., 1986). In addition, Carmo-Silva and Salvucci (2013) reported that the activity of Rubisco in *Arabidopsis thaliana* could remain a maximum of 30–50% in very low light intensity (<30 μmol photons m^–2^ s^–1^) (Carmo-Silva and Salvucci, 2013). Consequently, after the dark adaption, the Rubisco of leaves remain active, and dim light could partially activate the Rubisco in some species, hence, results in the partial induction of photosynthesis in the leaves of these species without light induction or with somewhat induction by dim light.

## Conclusion and Future Perspective

The PPFD of O_2_-evolving photolithotrophs on earth appears to be able to generate photosynthesis at 10 nmol photons m^–2^ s^–1^. Vascular plants have a similar photosynthetic process and equivalent energy demand. The numerous antenna pigments harvest photons and focus on one RC, and consequently generate the electronic potential for charge separation in vascular plant leaves. The fast and effective electron transfer prevents quenching and returning of the electron, which remains steady electron flux in the photosynthetic membrane. The electron can be accumulated for water-splitting through state S_0_ to S_4_, resulting in O_2_ evolving. Stomata, which may be different from photolithotrophs, cannot restraint gas exchange in the dim light, even if in darkness. The biochemical reaction Calvin cycle is also proved to be partially active in dim light. From the above, both reactions (dark reaction and light reaction) of photosynthesis can be conducted in dim light. Dim light occurs widely and lasts for a long time in natural and artificial environments, and this article showed that the photosynthesis of plant leaves could occur in this light condition. Thus, the increasing scenes of dim light might cause more contributions from the vascular plant to atmospheric carbon dioxide concentration on local or regional scales, which was closely related to plant development, crop yield, and climate change. In the future, the impact of dim light on plant photosynthesis should be investigated like normal light, and the models for estimation of crop yield and carbon budget should take dim light into consideration. The successful investigation to comprehend the utilization of dim light will require technological advancements to measure light characteristics and detecting methods to measure gas exchange at ecologically relevant levels in various field conditions, with theoretical foundations from this review. We hope that this article could provide some shreds of evidence for the research of carbon budget model, carbon sequestration, urban ecology, and understory ecology, etc.

## Author Contributions

XW did most of the data collection, XW, YS, HY, YW, AL wrote the first draft and ZG, FS, MA, QW, YS, RK edited and revised it. All authors contributed to the article and approved the submitted version.

## Conflict of Interest

YW and AL were both employed by Liangshan Branch of Sichuan Tobacco Company. The remaining authors declare that the research was conducted in the absence of any commercial or financial relationships that could be construed as a potential conflict of interest.

## References

[B1] Belgio E. Johnson M. P. Jurić S. Ruban A. V. (2012). Higher plant photosystem Ii light-harvesting antenna, not the reaction center, determines the excited-state lifetime-both the maximum and the nonphotochemically quenched *Biophys. J.* 102 2761–2771. 10.1016/j.bpj.2012.05.004 22735526 PMC3379028

[B2] Bennie J. Davies T. W. Cruse D. Gaston K. J. (2016). Ecological effects of artificial light at night on wild plants. *J. Ecol.* 104 611–620. 10.1111/1365-2745.12551

[B3] Blatt M. R. (2000). Cellular signaling and volume control in stomatal movements in plants. *Annu Rev. Cell Dev. Biol.* 16 221–241. 10.1146/annurev.cellbio.16.1.221 11031236

[B4] Breitler J. C. Djerrab D. Leran S. Toniutti L. Guittin C. Severac D. (2020). Full moonlight-induced circadian clock entrainment in coffea arabica. *BMC Plant Biol.* 20:24. 10.1186/s12870-020-2238-4 31941456 PMC6961272

[B5] Caemmerer S. Edmondson D. (1986). Relationship between steady-state gas exchange, in vivo ribulose bisphosphate carboxylase activity and some carbon reduction cycle intermediates in raphanus sativus. *Funct. Plant Biol.* 13 669–688. 10.1071/pp9860669

[B6] Caffarri S. Broess K. Croce R. Amerongen H. V. (2011). Excitation energy transfer and trapping in higher plant photosystem II complexes with different antenna sizes. *Biophys. J.* 100 2094–2103. 10.1016/j.bpj.2011.03.049 21539776 PMC3149253

[B7] Carmo-Silva A. E. Salvucci M. E. (2013). The regulatory properties of rubisco activase differ among species and affect photosynthetic induction during light transitions. *Plant Physiol.* 161 1645–1655. 10.1104/pp.112.213348 23417088 PMC3613445

[B8] Croce R. Van Amerongen H. (2014). Natural strategies for photosynthetic light harvesting. *Nat. Chem. Biol.* 10 492. 10.1038/nchembio.1555 24937067

[B9] Davies T. W. Smyth T. (2018). Why artificial light at night should be a focus for global change research in the 21st century. *Glob. Change Biol.* 24 872–882. 10.1111/gcb.13927 29124824

[B10] Dismukes G. C. Siderer Y. (1981). Intermediates of a polynuclear manganese center involved in photosynthetic oxidation of water. *Proc. Natl. Acad. Sci. U S A.* 78 274–278. 10.1073/pnas.78.1.274 16592949 PMC319035

[B11] Dubinsky Z. Schofield O. (2010). From the light to the darkness: thriving at the light extremes in the oceans. *Hydrobiologia* 639 153–171. 10.1007/s10750-009-0026-0

[B12] Ezequiel J. Laviale M. Frankenbach S. Cartaxana P. Serôdio J. (2015). Photoacclimation state determines the photobehaviour of motile microalgae: the case of a benthic diatom. *J. Exp. Mar. Biol. Ecol.* 468 11–20. 10.1016/j.jembe.2015.03.004

[B13] Gaston K. J. Bennie J. Davies T. W. Hopkins J. (2013). The ecological impacts of nighttime light pollution: a mechanistic appraisal. *Biol. Rev.* 88 912–927. 10.1111/brv.12036 23565807

[B14] Gerrish G. A. Morin J. G. Rivers T. J. Patrawala Z. (2009). Darkness as an ecological resource: the role of light in partitioning the nocturnal niche. *Oecologia* 160 525–536. 10.1007/s00442-009-1327-8 19330516

[B15] Grulke N. E. Alonso R. Nguyen T. Cascio C. Dobrowolski W. (2004). Stomata open at night in pole-sized and mature ponderosa pine: implications for O_3_ exposure metrics. *Tree Physiol.* 24 1001–1010. 10.1093/treephys/24.9.1001 15234897

[B16] Han Q. Yamaguchi E. Odaka N. Kakubari Y. (1999). Photosynthetic induction responses to variable light under field conditions in three species grown in the gap and understory of a fagus crenata forest. *Tree Physiol.* 19 625–634. 10.1093/treephys/19.10.625 12651318

[B17] Haumann M. Junge W. (1994). Extent and rate of proton release by photosynthetic water oxidation in thylakoids: electrostatic relaxation versus chemical production. *Biochemistry* 33 864–872. 10.1021/bi00170a003 8305433

[B18] Hoshika Y. Osada Y. De Marco A. Peñuelas J. Paoletti E. (2018). Global diurnal and nocturnal parameters of stomatal conductance in woody plants and major crops. *Glob. Ecol. Biogeogr.* 27 257–275. 10.1111/geb.12681

[B19] Joliot P. (2003). Period-four oscillations of the flash-induced oxygen formation in photosynthesis. *Photosyn. Res.* 76 65–72. 10.1023/A:1024946610564 16228566

[B20] Joliot P. Barbieri G. Chabaud R. (1969). A new model of photochemical centers in system-2. *Photochem. Photobiol.* 10 309–329.

[B21] Julian I. Schroeder, Gethyn J. Allen, Veronique Hugouvieux, June M. (2001). Guard cell signal transduction. *Annu. Rev. Plant Physiol. Plant Mol. Biol.* 52 627–658. 11337411 10.1146/annurev.arplant.52.1.627

[B22] Kaufmann M. R. (1976). Stomatal response of engelmann spruce to humidity, light, and water stress. *Plant Physiol.* 57 898–901. 10.1104/pp.57.6.898 16659594 PMC542144

[B23] Kirschbaum M. U. Ohlemacher C. Küppers M. (2004). Loss of quantum yield in extremely low light. *Planta* 218 1046–1053. 10.1007/s00425-003-1186-1 14722771

[B24] Kok B. Forbush B. Mcgloin M. (1970). Cooperation of charges in photosynthetic O_2_ evolution-I. A linear four step mechanism. *Photochem. Photobiol.* 11 457–475. 10.1111/j.1751-1097.1970.tb06017.x 5456273

[B25] Kouřil R. Nosek L. Semchonok D. Boekema E. J. Ilík P. (2018). “Organization of Plant Photosystem II and Photosystem I Supercomplexes,” in *Membrane Protein Complexes: Structure and Function*, eds Harris J. R. Boekema E. J. (Singapore: Springer), 259–286. 10.1007/978-981-10-7757-9_9 29464563

[B26] Kura-Hotta M. Hashimoto H. Satoh K. Katoh S. (1990). Quantitative determination of changes in the number and size of chloroplasts in naturally senescing leaves of rice seedlings. *Plant Cell Physiol.* 31 33–38.

[B27] Lawson T. (2009). Guard cell photosynthesis and stomatal function. *New Phytol.* 181 13–34. 10.1111/j.1469-8137.2008.02685.x 19076715

[B28] Lee J. S. (2010). Stomatal opening mechanism of CAM plants. *J. Plant Biol.* 53 19–23. 10.1007/s12374-010-9097-8

[B29] Mayoral O. Solbes J. Canto J. Pina T. (2020). What has been thought and taught on the lunar influence on plants in agriculture? perspective from physics and biology. *Agronomy* 10:955 10.3390/agronomy10070955

[B30] Mcevoy J. P. Brudvig G. W. (2006). Water-splitting chemistry of photosystem II. *Chem. Rev.* 106 4455–4483. 10.1021/cr0204294 17091926

[B31] Meidner H. Mansfield T. (1965). Stomatal responses to illumination. *Biol. Rev.* 40 483–508. 10.1111/j.1469-185x.1965.tb00813.x

[B32] Melis A. Neidhardt J. Benemann J. R. (1998). Dunaliella salina (Chlorophyta) with small chlorophyll antenna sizes exhibit higher photosynthetic productivities and photon use efficiencies than normally pigmented cells. *J. Appl. Phycol.* 10 515–525.

[B33] Meravi N. Kumar Prajapati S. (2020). Effect street light pollution on the photosynthetic efficiency of different plants. *Biol. Rhythm Res.* 51 67–75. 10.1080/09291016.2018.1518206

[B34] Mercado L. M. Bellouin N. Sitch S. Boucher O. Huntingford C. Wild M. (2009). Impact of changes in diffuse radiation on the global land carbon sink. *Nature* 458 1014. 10.1038/nature07949 19396143

[B35] Ogle K. Lucas R. W. Bentley L. P. Cable J. M. Barron-Gafford G. A. Griffith A. (2012). Differential daytime and night-time stomatal behavior in plants from north american deserts. *New Phytol.* 194 464–476. 10.1111/j.1469-8137.2012.04068.x 22348404

[B36] Ono K. Hashimoto H. Katoh S. (1995). Changes in the number and size of chloroplasts during senescence of primary leaves of wheat grown under different conditions. *Plant Cell Physiol.* 36 9–17.

[B37] Osterhout W. J. V. Haas A. (1918). On the dynamics of photosynthesis. *J. Gen. Physiol.* 1 1–16. 10.1007/0-306-48148-0_1 19871720 PMC2140291

[B38] Pearcy R. W. (1983). The light environment and growth of C_3_ and C_4_ tree species in the understory of a hawaiian forest. *Oecologia* 58 19–25. 10.1007/bf00384537 28310642

[B39] Pearcy R. W. (1988). Photosynthetic utilisation of lightflecks by understory plants. *Funct. Plant Biol.* 15 223–238. 10.1071/pp9880223

[B40] Pearcy R. W. Osteryoung K. Calkin H. W. (1985). Photosynthetic responses to dynamic light environments by hawaiian trees time course of CO_2_ uptake and carbon gain during sunflecks. *Plant Physiol.* 79 896–902. 10.1104/pp.79.3.896 16664512 PMC1074991

[B41] Putnam-Evans C. Barry B. A. (2007). Preface for special issue of photosynthesis research “photosynthetic water oxidation”. *Photosyn. Res.* 92 273–274. 10.1007/s11120-007-9226-5

[B42] Quigg A. Beardall J. Wydrzynski T. (2003). Photoacclimation involves modulation of the photosynthetic oxygen-evolving reactions in dunaliella tertiolecta and phaeodactylum tricornutum. *Funct. Plant Biol.* 30 301–308. 10.1071/fp02140 32689012

[B43] Raven J. Cockell C. (2006). Influence on photosynthesis of starlight, moonlight, planetlight, and light pollution (reflections on photosynthetically active radiation in the universe). *Astrobiology* 6 668–675. 10.1089/ast.2006.6.668 16916290

[B44] Raven J. Kübler J. Beardall J. (2000). Put out the light, and then put out the light. *J. Mar. Biol. Assoc. U K.* 80 1–25. 10.1017/s0025315499001526

[B45] Resco De Dios V. Chowdhury F. I. Granda E. Yao Y. Tissue D. T. (2019). Assessing the potential functions of nocturnal stomatal conductance in C_3_ and C_4_ plants. *N. Phytol.* 223 1696–1706. 10.1111/nph.15881 31055839

[B46] Retkute R. Smith-Unna S. E. Smith R. W. Burgess A. J. Jensen O. E. Johnson G. N. (2015). Exploiting heterogeneous environments: does photosynthetic acclimation optimize carbon gain in fluctuating light? *J. Exp. Bot.* 66 2437–2447. 10.1093/jxb/erv055 25788730 PMC4629418

[B47] Salisbury F. B. (1981). Twilight effect: initiating dark measurement in photoperiodism of *xanthium*. *Plant Physiol.* 67 1230–1238. 10.1104/pp.67.6.1230 16661842 PMC425867

[B48] Salvucci M. E. Portis A. R. Ogren W. L. (1986). Light and CO_2_ response of ribulose-1, 5-bisphosphate carboxylase/oxygenase activation in arabidopsis leaves. *Plant Physiol.* 80 655–659. 10.1104/pp.80.3.655 16664680 PMC1075178

[B49] Schulte M. Offer C. Hansen U. (2003). Induction of CO_2_-gas exchange and electron transport: comparison of dynamic and steady-state responses in fagus sylvatica leaves. *Trees* 17 153–163. 10.1007/s00468-002-0219-x

[B50] Shimazaki K.-I. Doi M. Assmann S. M. Kinoshita T. (2007). Light regulation of stomatal movement. *Annu. Rev. Plant Biol.* 58 219–247. 17209798 10.1146/annurev.arplant.57.032905.105434

[B51] Singhal R. K. Kumar M. Bose B. (2019). Eco-physiological responses of artificial night light pollution in plants. *Russ. J. Plant Physiol.* 66 190–202. 10.1134/s1021443719020134

[B52] Valladares F. Gianoli E. Saldaña A. (2011). Climbing plants in a temperate rainforest understorey: searching for high light or coping with deep shade? *Ann. Bot.* 108 231–239. 10.1093/aob/mcr132 21685433 PMC3143042

[B53] Van Bezouwen L. S. Caffarri S. Kale R. S. Kouřil R. Thunnissen A.-M. W. H. Oostergetel G. T. (2017). Subunit and chlorophyll organization of the plant photosystem II supercomplex. *Nature Plants* 3:17080. 10.1038/nplants.2017.80 28604725

[B54] Von Caemmerer S. (2000). *Biochemical models of leaf photosynthesis*. Clayton: Csiro publishing.

[B55] Wang Y. Noguchi K. Terashima I. (2008). Distinct light responses of the adaxial and abaxial stomata in intact leaves of helianthus annuus L. *Plant Cell Environ.* 31 1307–1316. 10.1111/j.1365-3040.2008.01843.x 18537998

[B56] Yamori W. Kusumi K. Iba K. Terashima I. (2020). Increased stomatal conductance induces rapid changes to photosynthetic rate in response to naturally fluctuating light conditions in rice. *Plant Cell Environ.* 43 1230–1240. 10.1111/pce.13725 31990076

