# Supplementary material for: Rationale: Photosynthesis of Vascular Plants in Dim Light
Source: Front Plant Sci. 2020 Nov 23;11:573881. doi: 10.3389/fpls.2020.573881 (PMC7732443; doi:10.3389/fpls.2020.573881)
Supplement: Supplementary file 1 [file Table_1.DOCX]

Supplementary Material

**Rationale: Photosynthesis of vascular plant in the dim light**

**Xiaolin Wang,^a^ Zhen Guo^b^, Qing Wang^c^, Rayyan Khan^a^, Huifeng Yan *^a^, Yi Shi*^a^**

a Tobacco Research Institute of Chinese Academy of Agricultural Sciences, Qingdao, PR China

b First Institute of Oceanography, Ministry of Natural Resources, Qingdao, PR China

c College of Tropical Crops, Hainan University, Haikou, PR China

**Reference for Figure 1:**

Belgio, E., Matthew P. Johnson, S. Jurić, and Alexander V. Ruban. 2012. Higher Plant Photosystem II Light-Harvesting Antenna, Not the Reaction Center, Determines the Excited-State Lifetime—Both the Maximum and the Nonphotochemically Quenched. Biophysical Journal **102**:2761-2771.

Caemmerer, S., and D. Edmondson. 1986. Relationship between steady-state gas exchange, in vivo ribulose bisphosphate carboxylase activity and some carbon reduction cycle intermediates in Raphanus sativus. Functional Plant Biology **13**:669-688.

Caffarri, S., K. Broess, R. Croce, and H. van Amerongen. 2011. Excitation Energy Transfer and Trapping in Higher Plant Photosystem II Complexes with Different Antenna Sizes. Biophysical Journal **100**:2094-2103.

Carmo-Silva, A. E., and M. E. Salvucci. 2013. The regulatory properties of Rubisco activase differ among species and affect photosynthetic induction during light transitions. Plant Physiology **161**:1645-1655.

Croce, R., and H. van Amerongen. 2014. Natural strategies for photosynthetic light harvesting. Nature Chemical Biology **10**:492.

de Dios, V. R., M. E. Loik, R. Smith, M. J. Aspinwall, and D. T. Tissue. 2016. Genetic variation in circadian regulation of nocturnal stomatal conductance enhances carbon assimilation and growth. Plant Cell and Environment **39**:3-11.

de Dios, V. R., J. Roy, J. P. Ferrio, J. G. Alday, D. Landais, A. Milcu, and A. Gessler. 2015. Processes driving nocturnal transpiration and implications for estimating land evapotranspiration. Scientific Reports **5**:8.

Dismukes, G. C., and Y. Siderer. 1981. Intermediates of a polynuclear manganese center involved in photosynthetic oxidation of water. Proceedings of the National Academy of Sciences **78**:274-278.

Haumann, M., and W. Junge. 1994. Extent and rate of proton release by photosynthetic water oxidation in thylakoids: electrostatic relaxation versus chemical production. Biochemistry **33**:864-872.

Hoshika, Y., Y. Osada, A. de Marco, J. Peñuelas, and E. Paoletti. 2018. Global diurnal and nocturnal parameters of stomatal conductance in woody plants and major crops. Global Ecology and Biogeography **27**:257-275.

Kok, B., B. Forbush, and M. McGloin. 1970. Cooperation of charges in photosynthetic O2 evolution–I. A linear four step mechanism. PHOTOCHEMISTRY AND PHOTOBIOLOGY **11**:457-475.

Kouřil, R., L. Nosek, D. Semchonok, E. J. Boekema, and P. Ilík. 2018. Organization of Plant Photosystem II and Photosystem I Supercomplexes. Pages 259-286 *in* J. R. Harris and E. J. Boekema, editors. Membrane Protein Complexes: Structure and Function. Springer Singapore, Singapore.

McEvoy, J. P., and G. W. Brudvig. 2006. Water-Splitting Chemistry of Photosystem II. Chemical Reviews **106**:4455-4483.

Quigg, A., J. Beardall, and T. Wydrzynski. 2003. Photoacclimation involves modulation of the photosynthetic oxygen-evolving reactions in *Dunaliella tertiolecta* and *Phaeodactylum tricornutum*. Functional Plant Biology **30**:301-308.

Resco de Dios, V., F. I. Chowdhury, E. Granda, Y. Yao, and D. T. Tissue. 2019. Assessing the potential functions of nocturnal stomatal conductance in C3 and C4 plants. New phytologist **223**:1696-1706.

Salvucci, M. E., A. R. Portis, and W. L. Ogren. 1986. Light and CO2 response of ribulose-1, 5-bisphosphate carboxylase/oxygenase activation in Arabidopsis leaves. Plant Physiology **80**:655-659.

Stirbet, A., and Govindjee. 2011. On the relation between the Kautsky effect (chlorophyll a fluorescence induction) and Photosystem II: Basics and applications of the OJIP fluorescence transient. Journal of Photochemistry and Photobiology B: Biology **104**:236-257.

Tamang, B. G., and W. Sadok. 2018. Nightly business: Links between daytime canopy conductance, nocturnal transpiration and its circadian control illuminate physiological trade-offs in maize. Environmental and Experimental Botany **148**:192-202.

van Bezouwen, L. S., S. Caffarri, R. S. Kale, R. Kouřil, A.-M. W. H. Thunnissen, G. T. Oostergetel, and E. J. Boekema. 2017. Subunit and chlorophyll organization of the plant photosystem II supercomplex. Nature Plants **3**:17080.

Von Caemmerer, S. 2000. Biochemical models of leaf photosynthesis. Csiro publishing.
